# Accounting for overlapping annotations in genomic prediction models of complex traits

**DOI:** 10.1186/s12859-022-04914-5

**Published:** 2022-09-06

**Authors:** Fanny Mollandin, Hélène Gilbert, Pascal Croiseau, Andrea Rau

**Affiliations:** 1 INRAE, AgroParisTech, GABI, Université Paris-Saclay, Allée de Vilvert, 78350 Jouy-en-Josas, France; 2 GenPhySE, INRAE, ENVT, Université de Toulouse, 31320 Castanet Tolosan, France; 3 BioEcoAgro Joint Research Unit, INRAE, Université de Liège, Université de Lille, Université de Picardie Jules Verne, 50136 Estrée-Mons, France

**Keywords:** Genomic prediction, Functional annotation, Bayesian models

## Abstract

**Background:**

It is now widespread in livestock and plant breeding to use genotyping data to predict phenotypes with genomic prediction models. In parallel, genomic annotations related to a variety of traits are increasing in number and granularity, providing valuable insight into potentially important positions in the genome. The BayesRC model integrates this prior biological information by factorizing the genome according to disjoint annotation categories, in some cases enabling improved prediction of heritable traits. However, BayesRC is not adapted to cases where markers may have multiple annotations.

**Results:**

We propose two novel Bayesian approaches to account for multi-annotated markers through a cumulative (BayesRC+) or preferential (BayesRC$\pi$) model of the contribution of multiple annotation categories. We illustrate their performance on simulated data with various genetic architectures and types of annotations. We also explore their use on data from a backcross population of growing pigs in conjunction with annotations constructed using the PigQTLdb. In both simulated and real data, we observed a modest improvement in prediction quality with our models when used with informative annotations. In addition, our results show that BayesRC+ successfully prioritizes multi-annotated markers according to their posterior variance, while BayesRC$\pi$ provides a useful interpretation of informative annotations for multi-annotated markers. Finally, we explore several strategies for constructing annotations from a public database, highlighting the importance of careful consideration of this step.

**Conclusion:**

When used with annotations that are relevant to the trait under study, BayesRC$\pi$ and BayesRC+ allow for improved prediction and prioritization of multi-annotated markers, and can provide useful biological insight into the genetic architecture of traits.

**Supplementary Information:**

The online version contains supplementary material available at 10.1186/s12859-022-04914-5.

## Background

It is now widespread in plant and animal breeding [1] and agriculture [2, 3] to predict phenotypes, i.e., observable traits in an individual, from genotypes. In recent years, improvements in sequencing technologies and their decreasing cost [4], combined with increased computational and storage capacities, have further accelerated the use of genomic prediction. Since the early 2000’s, a variety of genomic prediction models have been proposed, including the genomic best linear unbiased predictor (GBLUP; [5]) and the family of methods constituting the “Bayesian alphabet” [6], such as BayesA [1], BayesB [1], BayesC$\pi$ [7] and BayesR [8]. These models rely on different assumptions on the distribution of single nucleotide polymorphism (SNP) effects, striking a balance between flexibility and computational efficiency. BayesR in particular has been shown to be a powerful and flexible model, generally yielding high quality predictions while simultaneously facilitating quantitative trait loci (QTL) mapping, though some marker effects remain underestimated, especially for traits with low heritabilities [9, 10].

One interesting strategy for improvement to guide genomic prediction models is the use of prior biological information [11]. An increasing amount of such prior information is available, ranging from publicly available trait mapping information to functional or structural annotations of the genome [12]. By appropriately including these heterogeneous and complex data, it is hoped that causal mutations could be more readily identified and prioritized, thus potentially improving model predictions and interpretability. Such prior biological information can be collected on the same individuals used for genomic prediction, or on an independent population (e.g., from publicly available databases, such as FAANG “Functional Annotation of Animal Genome” [12]). Several methods have been introduced for the former case, such as GTBLUP [13] and GTiBLUP [14], although such fully coupled datasets remain rare. However, prior biological information from independent sources are much more widely accessible, and such information can be used to assign variants to annotation categories.

To make use of such information, the BayesRC model [15] factorizes the genome according to a prior categorization of markers. Each annotation category is independently modeled according to the mixture prior defined by BayesR, where SNPs may have a null, small, medium or large effect. In practice, BayesRC is generally used for a small number of disjoint annotation categories, where each marker is assigned to a single category. Considering a greater number of potentially overlapping annotations would likely lead to the presence of multi-annotated markers. To use BayesRC in this case would necessitate the choice of a single annotation for each multi-annotated marker, which may lead to an undesired loss of information. There thus remains a need to define robust models that can handle multi-annotated SNPs.

Depending on the context, choice of annotations, and desired interpretation of multi-annotated SNPs, in this work we propose two different models. First, if multiple annotation categories are thought to represent ambiguity in the appropriate annotation assignment, we directly model the probability of category assignment for multi-annotated SNPs. For this, we propose the BayesRC$\pi$ model with a mixture of mixtures prior distribution on SNP effects, thus allowing multi-annotated SNPs to be assigned a posteriori to the most informative annotation. Alternatively, if the number of annotations for a given marker is assumed to be informative (e.g., a larger number of annotations implies a stronger belief that a marker may be causal), we may wish to systematically assign greater weight to multi-annotated markers in the model. To this end, we propose the BayesRC+ model, with a cumulative mixture prior distribution on SNP effects.

As the proposed BayesRC$\pi$ and BayesRC+ models incorporate biological information in different ways, their performance is likely to be highly dependent on the underlying genetic architecture of the studied traits, the construction of annotation categories, and the biological relevance of the prior information. However, given the potential difficulty in defining annotation categories in practice, both models must be robust to the inclusion of noisy or irrelevant annotations. To evaluate our models, we simulated data with various genetic architectures and annotation configurations. In the same perspective, we applied both methods to real pig data in conjunction with annotations constructed from a public database, again varying the way annotation categories were constructed. This study thus highlights the interest of BayesRC$\pi$ and BayesRC+ in different scenarios, while proposing a preliminary, non-exhaustive framework for their use in practice.

## Methods

### Bayesian genomic prediction without annotations

The general statistical model for genomic prediction can be defined as

1 $$\begin{aligned} {\textbf {y}}&=\mu \mathbbm {1}_n+{\textbf {X}}\varvec{\beta }+{\textbf {e}},\nonumber \\ {\textbf {e}}&\sim N(0,{\textbf {I}}_{n}\sigma ^{2}_{e}) \end{aligned}$$

where $\mathbf {y}$ is the vector of corrected phenotypes, $\mu$ the intercept, $\varvec{\beta }$ the vector of the SNP effects and $\mathbf {e}$ the vector of residuals. We assume that $\mathbf {e}$ follows a multivariate normal distribution with mean 0 and variance covariance matrix $\mathbf {I}_n\sigma ^2_e$. $\mathbf {X}$ is the marker matrix, centered and scaled such that $X_{ij}=(w_{ij}-2f_j)/\sqrt{2f_j(1-f_j)}$, with $w_{ij}\in \{0,1,2\}$ the number of copies of the alternative allele of marker *j* in individual *i* and $f_j$ the frequency of the alternative allele of marker *j* in the full population. We note $\sigma _g^2$ the total additive variance, i.e., the cumulative variance of all SNP effects.

By defining different prior distributions on the SNP effect vector $\varvec{\beta }$, models in the Bayesian alphabet seek to overcome model overparametrization in various ways. BayesR [8] assumes that SNP effects $\beta _i$ follow a four-component normal mixture:

2 $$\begin{aligned} f(\beta _i)&= \sum _{k=1}^4 \pi _k f_k(\cdot \vert \theta _k) \end{aligned}$$

3 $$\begin{aligned} {\text {such}}\,{\text {that}}\,f_k&= \left\{ \begin{array}{ll} \delta (0), &{}{\text {if}}\,\,k=1 \\ \phi (\cdot \vert 0,\theta _k) &{}{\text {otherwise}} \end{array} \right. \end{aligned}$$

where $\varvec{\theta }=(\theta _2,\theta _3,\theta _4)=(0.0001\sigma _g^2,0.001\sigma _g^2,0.01\sigma _g^2)$, $\sum _{k=1}^4\pi _k=1$, $\delta (0)$ represents a point mass at 0, and $\phi$ is the centered Gaussian probability density function.

Practically, the BayesR model implies that markers are assigned to one of four different effect size classes: null, small, medium or large, corresponding respectively to 0%, 0.01%, 0.1% and 1% of the total additive genetic variance $\sigma _g^2$. The mixing proportions $\varvec{\pi }=(\pi _1,\pi _2,\pi _3,\pi _4)$ are assumed to follow a Dirichlet prior, corresponding to the posterior $f(\varvec{\pi } \vert .) \sim \mathrm {Dirichlet}(\varvec{\alpha }+\varvec{\gamma })$, with $\varvec{\alpha }$ representing a vector of pseudocounts and $\varvec{\gamma }$ the cardinality of each component. In this work, we used a flat Dirichlet pior distribution, with $\varvec{\alpha }=(1,1,1,1)$, and $\sigma _g^2$ is assumed to be a random variable following an $\mathrm {Inv-}\chi ^2$ distribution.

### Formalizing annotation categories

There are several potential ways that biological annotations could be formalized for use as prior information. Here, we assume that markers are categorized in a binary fashion for each category (annotated or not), where SNPs with no known annotation are aggregated together under an “other” category. We note $C_i \subseteq \{c_1,c_2,...,c_m\}$ the set of annotations corresponding to SNP *i*. Depending on the case, marker *i* can have a single annotation (i.e., $\vert C_i \vert =1$) or be multi-annotated (i.e. $\vert C_i\vert \ge 1$).

### Bayesian genomic prediction with disjoint annotations

BayesRC [15] extends the BayesR model prior in Eq. (3) by dividing the genome into disjoint annotations such that $\vert C_i \vert =1$, each with a potentially different proportion of small, medium, and large QTLs. BayesRC thus exploits the same four SNP effect size classes as BayesR, but the mixing proportions $\varvec{\pi }_c$ for each annotation *c* are estimated separately:

$$\begin{aligned} f(\beta _i \vert \mathbf {C}_i=c)&= \sum _{k=1}^4 \pi _{k,c} f_k(\cdot \vert \theta _k), \\ {\text {such}}\,{\text {that}}\, f_k&= \left\{ \begin{array}{ll} \delta (0), &{}{\text {if}}\,k=1 \\ \phi (\cdot \vert 0,\theta _k)&{} {\text {otherwise}} \end{array} \right. \end{aligned}$$

where $\varvec{\theta }$, $\delta (0)$, and $\phi$ are defined as before, and $\sum _{k=1}^4\pi _{k,c}=1$ for all $c \in \{c_1,c_2,...,c_m\}$. The mixing proportions $\varvec{\pi }_{c}$ are assumed to follow a Dirichlet prior, yielding the posterior $f(\varvec{\pi }_{c} \vert . ) \sim \mathrm {Dirichlet}(\varvec{\alpha }+\varvec{\gamma }_{c})$, with $\varvec{\alpha }$ representing a vector of pseudocounts and $\varvec{\gamma }_{c}$ the cardinality of each component in annotation *c*. As for BayesR, we used a flat Dirichlet distribution, with $\varvec{\alpha }=(1,1,1,1)$, for the mixing proportion priors. In order to limit the impact this prior can have on the posterior, MacLeod et al. [15] recommend using relatively common annotations (i.e., including more than about 1000 markers).

### Bayesian genomic prediction with overlapping annotations

By increasing the number and variety of (potentially redundant) annotations, it becomes increasingly likely to have multi-annotated markers where $\vert C_i\vert \ge 1$ for some *i*. By definition, BayesRC cannot directly account for such overlapping annotations, limiting the full use of available information. To address this, we propose two novel methods for exploiting overlapping annotations in different contexts: BayesRC$\pi$ and BayesRC+.

#### BayesRC$\pi$

In cases where multiple annotation categories include potential ambiguity for multi-annotated SNPs, it may be of interest to model the probability of category assignment. To this end, we propose the BayesRC$\pi$ model to allow multi-annotated markers to preferentially associate with annotations, according to their coherence with the respective SNP effect distributions. Specifically, we define a mixture of mixtures prior distribution for SNP effects:

$$\begin{aligned} f(\beta _i \vert \mathbf {C}_i)&= \sum _{c \in \mathbf {C}_i} p_{i,c}\sum _{k=1}^4 \pi _{k,c} f_k(\cdot \vert \theta _k) \\ {\text {such}}\,{\text {that}}\,f_k&= \left\{ \begin{array}{ll} \delta (0), &{}{\text {if}}\, k=1 \\ \phi (\cdot \vert 0,\theta _k)&{} {\text {otherwise}} \end{array} \right. \end{aligned}$$

where $\varvec{\theta }$, $\delta (0)$, and $\phi$ are defined as before and $\sum _{k=1}^4\pi _{k,c}=1$ for all $c \in \{c_1,c_2,...,c_m\}$. We have thus introduced the mixing parameter $\mathbf {p}_i \in ]0,1]^{\vert C_i \vert }$ for SNP *i* in its set of annotations $\mathbf {C}_i$, such that $\sum _{c \in {\mathbf {C}_i}}p_{i,c}=1$ for all *i*. Once again, the mixing proportions $\varvec{\pi }_{c}$ are assumed to follow a Dirichlet prior, giving the posterior $f(\varvec{\pi }_c \vert .)\sim \mathrm {Dirichlet}(\varvec{\alpha }+\varvec{\gamma }_{c})$, with $\varvec{\alpha }=(1,1,1,1)$. The mixing proportions $\mathbf {p}_i$ are assumed to follow a Dirichlet prior, with size depending on the cardinality of the annotation set of each SNP *i*.

#### BayesRC+

An alternative way of interpreting a multi-annotated marker is to assume that a greater number annotations implies that more weight should be attributed to the marker in the model. In this spirit, we propose the BayesRC+ model to assign an additive impact of multiple annotation categories on estimated SNP effects. Multi-annotated variants will thus tend to have a greater chance to be included as non-null in the model, and as such a larger estimated effect. Specifically, we define a cumulative mixture prior distribution for the effect of SNP *i*:

$$\begin{aligned} f(\beta _i \vert {\textbf {C}}_i)&= \sum _{c \in {\textbf {C}}_i}\sum _{k=1}^4 \pi _{k,c} f_k(\cdot \vert \theta _k) \\ {\text {such}}\,{\text {that}}\,f_k&= \left\{ \begin{array}{ll} \delta (0), &{}{\text {if}}\, k=1 \\ \phi (\cdot \vert 0,\theta _k)&{} {\text {otherwise}} \end{array} \right. \end{aligned}$$

where $\varvec{\theta }$, $\delta (0)$ and $\phi$ are defined as before and $\sum _{k=1}^4\pi _{k,c}=1$ for all $c \in \{c_1,c_2,...,c_m\}$. Prior and posterior distributions for the mixing proportions $\varvec{\pi }_{c}$ are as described above in the BayesRC and BayesRC$\pi$ models.

### Gibbs sampling

As the full posterior distributions for all models described above are intractable, model parameters are estimated with a Gibbs sampler, using the posterior mean of their estimations. Algorithm implementation details for BayesR were previously described by Kemper et al. [16] and Moser et al. [9] and were used as a base to implement BayesRC, BayesRC$\pi$ and BayesRC+. Broad steps of the algorithms for each are shown in pseudocode Additional file 1: Algorithms 1–4.

Concretely for BayesRC$\pi$, within a given iteration of the Gibbs sampler SNPs are assigned to the annotation category with probability proportional to its conditional likelihood given the current estimates of other model parameters. Note that this step is analogous to that in the standard BayesR algorithm of assigning SNPs to one of the four effect classes, based on a conditional likelihood calculation given the current estimates of model parameters. For BayesRC+, at each iteration of the Gibbs sampler the conditional effect of a given SNP is estimated for each of its associated annotation categories in turn, and its total effect is subsequently calculated as the sum over all of its per-annotation effects.

For all models, we ran the Gibbs sampler algorithm for 50,000 iterations, discarding 20,000 for burning, and using a thinning rate of 10.

### *BayesRCO* package on Github

We propose the *BayesRCO* (BayesRC for Overlapping annotations) software, which implements five different Bayesian genomic prediction models, including three state-of-the art approaches (BayesC$\pi$ [7], BayesR, and BayesRC) and our two novel algorithms, BayesRC$\pi$ and BayesRC+. The implementation of our two new models builds on that of the *bayesR* software found at https://github.com/syntheke/bayesR [9]. Since BayesR can be seen as a special case of BayesRC with a single annotation category, and BayesRC as a special case of BayesRC$\pi$ and BayesRC+ using non-overlapping annotations, the *BayesRCO* algorithm is divided into three independent modules: BayesC$\pi$, BayesRC$\pi$ and BayesRC+.

### Metrics for evaluation

All the metrics defined here are used to evaluate the models on the simulated and real data defined in the next section.

#### Prediction accuracy

Prediction accuracy for all models was quantified using the Pearson correlation between the true ($\mathbf {y}$) and estimated ($\hat{\mathbf {y}}$) phenotypic values in the validation set.

#### Posterior variance

The posterior variance of SNP *i* can be estimated as:

$$\begin{aligned} \widehat{V_i}&=\widehat{\beta _i^2} Var(X_{.i}), \end{aligned}$$

where $X_{.i}$ represents the $i{\mathrm {th}}$ column of the centered and scaled genotype design matrix and $\widehat{\beta _i^2}$ corresponds to the posterior mean of $\beta _i^2$, estimated by $\widehat{\beta _i^2}=\frac{1}{N}\sum _{\ell =1}^N\beta _i^{(\ell )2}$, where *N* is the number of iterations and $\beta _i^{(\ell )2}$ the value of $\beta _i^{2}$ at iteration $\ell$. As the SNP effects are computed on the scaled and centered genotype design matrix *X*, the per-SNP posterior variance can be estimated using $\widehat{V_i}=\widehat{\beta _i^2}$.

#### Assignment of annotation categories using BayesRC$\pi$

The specificity of BayesRC$\pi$ is that it models the assignment of multi-annotated markers to different annotation categories. To quantify these assignments, we introduce the posterior annotation inclusion probability (PAIP), representing the frequency (across Gibbs sampler iterations) of assignment for each multi-annotated marker to each of its annotations. We note $\text {PAIP}_i=\{\text {PAIP}_{i,c_1},\ldots ,\text {PAIP}_{i,c_m}\}$ the PAIP of marker *i* such that, for all *i*, $\text {PAIP}_{i,c}\in \ ]0,1]$ for all $c\in {\textbf {C}}_i$, and $\sum _{c \in \mathbf {C}_i} \text {PAIP}_{i,c}=1$ for all *i*.

### Simulation framework

We next sought to simulate phenotypes associated with genomic data and associated annotations (as described below) to evaluate our models. For this purpose, we used real Illumina Bovine SNP50 BeadChip genotyping data from $n=2605$ Montbéliarde bulls. Using these data as a base for our simulations has the advantage of including realistic population and linkage disequilibrium (LD) structures in our simulations. We excluded SNPs with a minor allele frequency (MAF) less than 0.01, leaving a total of $p=46{,}178$ SNPs. We divided individuals into learning and validation sets, respectively consisting of 80% of the oldest (2083 bulls) and 20% of the youngest bulls (522 bulls).

#### Phenotype simulation

To simulate phenotypes $\mathbf {y}$ for the $n=2605$ bulls, we used the linear model in Equation (1), with parameters set as follows. For each simulated dataset, we randomly sampled a set of SNPs among those with a MAF $\ge 0.15$; by focusing on frequent variants, we sought to reduce the impact of extreme MAFs on genomic prediction [17]. For selected SNPs, the corresponding effect $\beta _i$ for selected SNP *i* was set as follows:

$$\begin{aligned} \beta _i=\frac{1}{2}u_i\sqrt{\frac{k\sigma ^2_g}{2\text {MAF}_i(1-\text {MAF}_i)}}, \end{aligned}$$

where $k \in \lbrace k_\text {small},k_\text {medium},k_\text {large}\rbrace$ corresponds to the proportion of the total additive variance $\sigma _g^2$ for a given effect size class (described below), MAF represents the frequency of the alternative allele in the population, and $u_i$ is drawn from a discrete $\mathrm {Uniform}\lbrace -1, 1\rbrace$ distribution to allow non-null effects to take on positive or negative values. For remaining (unselected) SNPs, $\beta _i$ was set to 0. Note that this ensures that the explained variance is the same for each simulated QTL regardless of its frequency. In addition, this guarantees that the sum of all explained variances per SNP is equal to the fixed total additive variance.

In all simulations, we selected a total of $n_\text {large}=5$ large QTLs, varying the corresponding proportion of the total genetic additive variance $\sigma _g^2$ such that $k_\text {large} \in \{1\%,2.5\%,5\%\}$. We also selected $n_\text {medium}=300$ medium QTLs, each representing $k_\text {medium}=0.1\%$ of $\sigma _g^2$. We filled in the remaining genetic additive variance with small effect SNPs representing $k_\text {small}=$0.01% of $\sigma _g^2$. The number of these small effect SNPs varied according to the chosen value of $k_\text {large}$, respectively corresponding to $n_\text {small}=\lbrace 6500,5750,4500\rbrace$. Finally, the phenotypic variance and mean were respectively set to $\sigma _y^2=100$ and $\mu =0$, and SNP heritability $h^2=\frac{\sigma _g^2}{\sigma _y^2}$ was set to one of two levels: $h^2=\{0.2,0.5\}$. For each simulation setting, 50 independent datasets were simulated.

#### Simulation of annotations

Annotations are defined here as informative when they are enriched in (i.e., contain a large proportion of) non-null markers, thus explaining a non-negligible portion of the total variance. To evaluate the impact of different annotation configurations on our models, we introduce four types of annotations: unenriched (i.e., uninformative), weakly enriched, moderately enriched and strongly enriched. Each annotation is constructed as shown in the upper half of Table 1 by randomly assigning different effect size SNPs (as well as their immediate neighbors). We note that each annotation constructed in this fashion contains around 1200–1300 markers.

**Table 1:** Simulation settings for the annotation scenarios

	Annotation enrichment			
	Strongly	Moderately	Weakly	Unenriched
SNP effect class				
Large	5	2	–	–
Medium	300	100	20	–
Low/null	150	300	400	450
Scenario				
A	1	1	–	–
B	1	1	1	1
C	–	2	1	1
D	2	2	3	2

We defined 4 levels of non-null SNP enrichment for simulated annotations (top part of the table) that can be mixed and matched to construct various scenarios (bottom part of the table). The top part shows the number of SNPs of each size effect class (rows) used to construct each level of annotation enrichment (columns). The bottom part indicates the number of each type of annotation enrichment (columns) used to construct each scenario (rows)

These individual annotations can then be mixed and matched to form (partially) overlapping annotation sets. To simulate scenarios with different combinations of annotations, these individual annotations were then mixed and matched to form (partially) overlapping annotation sets. We focus on 4 annotation scenarios defined in the lower half of Table 1. Scenario A consists of one strongly and one moderately enriched annotations. Scenario B builds on scenario A by adding noise via two less enriched annotations. Scenario C represents a potentially less advantageous case, with no strongly enriched annotation. Finally, scenario D combines nine annotations with varying levels of enrichments, thus creating more overlaps and greater ambiguity. Given the number of simulated total large, medium and low QTL effects (5, 300 and more than 4500, respectively, see previous section), many large and medium-effect QTLs are then multi-annotated in all the scenarios.

Recall that overlapping annotations cannot be exploited for BayesRC. To include it in our comparisons, we used a naive work-around for this issue to randomly select a single annotation for each multi-annotated marker for BayesRC. As such, annotations used for BayesRC in the following results are not quite the same as those used for BayesRC$\pi$ and BayesRC+.

### Production traits genomic prediction using QTL public database for growing pigs

#### Data description and pre-processing

A set of $n=634$ and $n=664$ animals (from 60 and 70 Large-White sows, respectively) from a population of 75% Large-White $\times$ 25% Creole crossbred pigs were raised in a temperate or tropical environment [18]. These offspring were descendants of a common batch of 10 boars that were themselves crossbred 50% Large-White $\times$ 50% Creole. A variety of traits were measured in this experiment using a common recording protocol in the two environments; trait measurements were pre-corrected for environment, age and sex effects. In this paper, we focus in particular on back fat thickness (BFT) and average daily weight gain (ADG), both measured at 23 weeks. For these traits, a total of $n=1147$ and $n=1146$ animals were respectively phenotyped. Animals were genotyped with the Illumina Porcine 60k BeadChip array.

To establish the potential impact of our models on prediction accuracy, we used a sibling-structured 10-fold cross validation procedure. For the descendants from each sire in turn, we calculated the correlation between their observed corrected phenotypes and those predicted from models constructed on the descendants of the remaining 9 sires; validation correlations were averaged across the ten folds. As the number of offspring per boar was relatively homogeneous, we thus obtained an approximate split of 90–10% between training and validation sets. Using PLINK [19], we filtered out genotypes with a MAF $< 0.01$ for each training set independently, and retained only markers across all ten training sets ($p=46,908$ and 4, 6881 SNPs for BFT and ADG, respectively).

#### Strategies for constructing annotations from pigQTLdb

Animal QTLdb (https://www.animalgenome.org/QTLdb) groups together curated results from genotype-phenotype association studies in several livestock species [20]. Cross-experiment QTL data from PigQTLdb (Release 45; SS11.1) for traits relevant to pig production were downloaded for eleven trait sub-hierarchy categories (anatomy, behavioral, blood parameters, conformation, fatness, fatty acid content, feed conversion, fowth, immune capacity, litter traits, reproductive organs). An additional “other” category was created for markers not included in PigQTLdb.

The potential utility of annotations depends on several factors, including their quality, relevance to the trait considered, LD around annotated mutations, and concordance in genotyping and annotation density (e.g., low density genotypes versus sequence-level information). An interesting strategy to consider is the use of expanded annotated windows around markers of interest, as well as the appropriate size of such a window; adding too many neighboring markers to annotations runs the risk of diluting the information they contribute. In this study, we explored three strategies for constructing annotations for genotyped markers: (1) using the exact position of known PigQTLdb markers (“regular”); (2) using the position of known PigQTLdb markers extended by a hard window, i.e., including the nearest up- and downstream neighbors (“hard”); and (3) using the position of known PigQTLdb markers as before but instead extended by a fuzzy window, where neighboring markers were allowed ambiguous assignment to both trait-specific and “other” categories (“fuzzy”). This latter strategy is particularly suited to BayesRC$\pi$, as it allows markers in the neighborhood of annotated SNPs the possibility or not of inclusion with the respective annotation. “Regular” and “hard” annotations were used with BayesRC (with downsampling as before to avoid multiple annotations), BayesRC$\pi$ and BayesRC+, while “fuzzy” annotations were used with BayesRC$\pi$ alone. In the three strategies, 1.3%, 4.9% and 17.7% of markers were respectively assigned to two or more categories (Additional file 1: Fig. S4). The same three sets of annotations were used for both the BFT and ADG traits.

## Results

### Simulation results

We evaluated our proposed BayesRC$\pi$ and BayesRC+ models, compared to BayesR (without annotations) and BayesRC (with downsampled annotations to remove overlaps). We focused on their predictive power, as well as their ability to prioritize true QTLs, multi-annotated markers, and informative annotation categories.

#### Impact of annotation scenarios on prediction accuracy

**Fig. 1:**
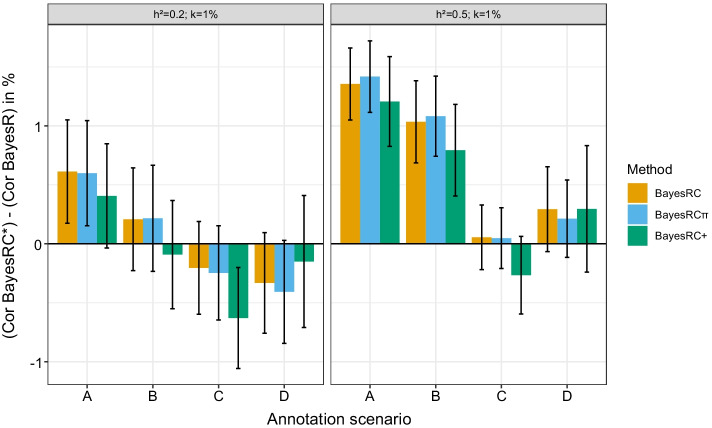
Differences in validation correlation with respect to BayesR for four annotation scenarios. For $h^2=0.2$ and $h^2=0.5$ and $k_\text {large}=1\%$, the difference in validation correlation between the three models including annotations (BayesRC, BayesRC$\pi$ and BayesRC+, gathered under the BayesRC* label) and BayesR, which does not include annotations. Colored bars and error bars represent averages and 95 % confidence interval across 50 simulated datasets

We calculated the Pearson correlation between simulated and estimated phenotypes in the validation data for each model in each simulation scenario (annotation configuration, heritability, large QTL effect size). Results were averaged across the 50 simulated datasets for each setting. As a baseline, we consider the results for BayesR, which ignores annotation information. Hence, we tested the difference of correlation for each of the following pairs of models: {BayesRC vs BayesR}, {BayesRC$\pi$ vs BayesR} and {BayesRC+ vs BayesR}. In the case of $h^2=0.2$, average (± sd) BayesR prediction accuracy was 0.211 (± 0.050), 0.224 (± 0.053) and 0.234 (± 0.054) for $k_\text {large}$ of 1%, 2.5% and 5%. For $h^2=0.5$, these values were respectively 0.447 (± 0.048), 0.464 (± 0.051) and 0.497 (± 0.045). As expected, we observed improved predictions with higher heritability and stronger QTL effects. However, as suggested by the 95% level confidence intervals shown in Fig. 1 and Additional file 1: Fig. S1, not all of these differences are significant (paired t-test at 95% level). In particular, if for scenarios A and B in most settings (except for $h^2$ and small *k*), one can conclude that the methods incorporating functional annotations significantly improve the correlation, this is not always the case for scenarios C and D. In general, the tests were significant in cases where graphically the confidence interval did not reach the x axis.

We next turn to the impact observed for models incorporating annotations. The average differences in correlation with respect to BayesR are shown for BayesRC, BayesRC$\pi$ and BayesRC+ for all settings with $k_\text {large}=1\%$ in Fig. 1. Results for $k_\text {large}$ = 2.5% and 5% are shown in the Additional file 1. First, we observe that incorporating annotations in BayesRC does not lead to a universal gain in prediction accuracy compared to BayesR across scenarios. For $h^2=0.2$, BayesRC gains on average 0.6 (±1.6) and 0.2 (±1.6) points for scenarios A and B, but loses 0.2 (±1.4) and 0.3 (±1.5) correlation points for scenarios C and D. With higher heritability ($h^2=0.5$), BayesRC leads to average gains of 1.6 (±1.1), 1.0 (±1.2), 0.1 (±1.0), and 0.3 (±1.3) for scenarios A, B, C, and D. BayesRC thus seems to perform best when the provided annotations are informative and contain little noise (scenario A). For larger QTL sizes (Additional file 1: Fig. S1), similar trends are observed, with potentially higher prediction gains (up to 2 correlation points for $h^2=0.2$, $k_\text {large}=5\%$ in scenario A). Moreover, a positive average gain is observed in all scenarios with BayesRC for sufficiently large QTLs and/or heritability.

We next looked at whether a better use of overlapping annotations could improve prediction accuracy. Overall, the differences in prediction between BayesRC and BayesRC$\pi$ are slight, corresponding to an average gain or loss of about 0.1 correlation points depending on the scenario and setting. At most, BayesRC$\pi$ led to a 1-point gain in correlation, for one dataset with $h^2=0.2$, $k_\text {large}=5\%$ and scenario A. The small differences here can likely be explained by the construction of annotation sets, where the random downsampling of annotations for BayesRC still tends to categorize multi-annotated markers in an enriched annotation, a favorable situation for BayesRC; as such markers are already well-ranked by BayesRC, the impact of BayesRC$\pi$ will be limited. On the other hand, the underlying additive hypothesis of BayesRC+ distinguishes it more from BayesRC, so we can expect to see larger differences. Once again, predictions are better with more informative annotation scenarios (A and B) than with the noisier annotation sets in scenarios C and D. BayesRC+ underperforms BayesRC for scenarios A, B and C, but shows better results in scenario D, reaching an average gain of 0.5 points for $h^2=0.2$ and $k_\text {large}=5\%$. BayesRC+ thus seems to be more robust to the addition of noise in annotations, given that there are some that are sufficiently informative. In the contrary case (scenario C), BayesRC+ risks too strongly prioritizing unimportant markers, thus deteriorating the prediction accuracy.

#### Model behavior for multi-annotated markers

BayesRC+ and BayesRC$\pi$ are designed to handle multi-annotated SNPs differently. With BayesRC$\pi$, we aim to reclassify multi-annotated markers to the annotation whose enrichment which best matches their estimated effect. This has the added advantage of providing useful information about the probability of assignment for each annotation across iterations of the Gibbs sampling algorithm (via the PAIP statistic). On the other hand, BayesRC+ assumes that multi-annotated markers should be more likely to have a non-null effect (and thus, potentially a higher variance) in the model, counterbalancing an underestimation of QTL effects.

**Fig. 2:**
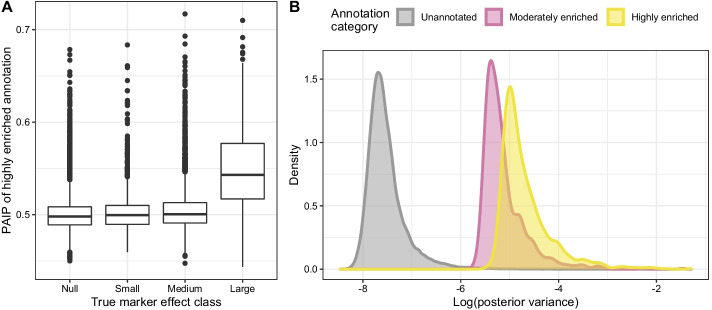
Using the PAIP to interpret annotation importance for BayesRC$\pi$. The PAIP, “Posterior Annotation Inclusion Probability”, is defined as the frequency of marker assignment in each annotation across iterations. Results are shown for $h^2=0.5$, $k_\text {large}=1\%$, and scenario A. **A** Posterior mean frequency of marker assignment to the strongly enriched annotation (i.e., strongly enriched PAIP) by simulated effect size category (null, small, medium, high). Results are averaged across 50 independent datasets. **B** Distribution of the log posterior variance of markers by PAIP-assigned annotation for one illustrative dataset

We focus on Scenario A here, as it provides the simplest illustration of model behavior for multi-annotated markers. In particular, it is constructed using only two annotations, one highly and one moderately enriched; as such, multi-annotated markers are necessarily included in both. In Fig. 2A, we represent the PAIP of the highly enriched annotation as a function of simulated marker size category ($h^2=0.5$, $k_\text {large}=1\%$). We clearly distinguish the large effect QTLs, which have an average highly enriched PAIP of 0.556. In fact, 89.4% of these large QTLs were predominantly assigned (PAIP $> 0.5$) to the highly enriched rather than the moderately enriched annotation. In comparison, we observe an average highly enriched PAIP of {0.505,0.502,0.500} for the medium, small and null marker effect sizes respectively, and a corresponding proportion of preferential assignment to the highly enriched annotation of 52.9%, 49.3% and 45.0%. This suggests that for QTLs with sufficiently large effects, using a maximum a posteriori (MAP) classification rule could provide useful insight into annotation enrichment. In Fig. 2B we show the densities of log posterior variance by assigned annotation category (via a MAP rule for the PAIP) for one representative dataset (scenario A, $h^2=0.5$, $k_\text {large}=1\%$). The distributions of $\log \widehat{V_i}$ are distinct for each category, though that of unannotated SNPs is clearly separated from those of the moderately and strongly enriched annotation categories. This seems to be consistent with the simulated distributions of annotations in scenario A, for which strong and medium QTLs were found in both annotations; larger differences in annotation enrichments may lead to a greater effect on PAIP values. For example, moving from $k_\text {large}=1\%$ to $k_\text {large}=5\%$, the share of $\sigma _g^2$ contributed by large QTLs in the strongly and moderately enriched annotations increases respectively from 5% to 25%, and from 2% to 10%. In this case, the strongly enriched PAIP for large QTLs increases drastically (to an average of 0.770) compared to the $k_\text {large}=1\%$ case (Additional file 1: Fig. S2). Larger QTL effects thus lead to higher values for the strongly enriched PAIP, and a systematic assignment (100%) of large QTLs to the strongly enriched annotation using a MAP rule for PAIPs. At the same time, this proportion decreases to 49.4%, 43.1% and 40.6% respectively for the medium, low and null categories of simulated markers.

**Fig. 3:**
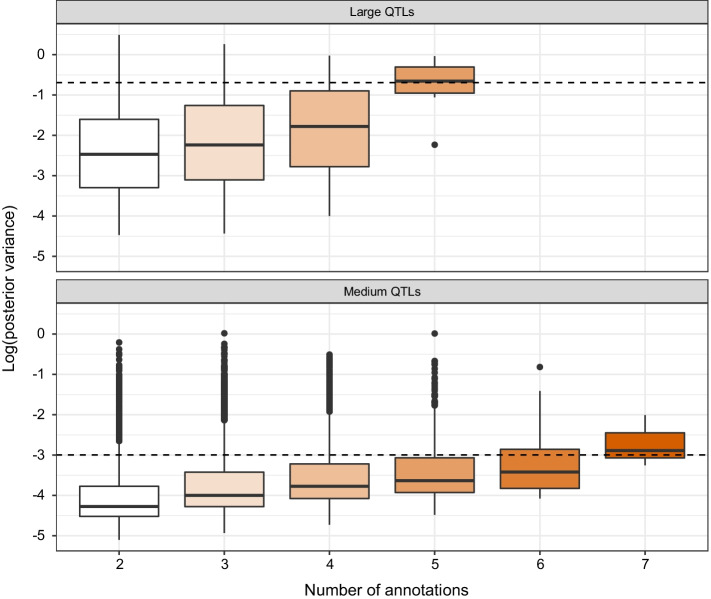
Impact of number of annotations on markers for BayesRC+ model. Log posterior variance of large (top panel) and medium (bottom panel) effect QTLs by the number of associated annotations. All QTLs across the 50 independent datasets are represented. Results are shown for $h^2=0.5$, $k_\text {large}=1\%$ and scenario D (including 9 annotations). The black dotted lines represent the true simulated value of $\log V_i$ for large and medium QTLs

With BayesRC+, we instead seek to explicitly prioritize multi-annotated markers; this prioritization depends both on the number and quality of annotations for each marker. We now turn our attention to Scenario D, which is composed of a set of 9 annotations. In Fig. 3, we represent the $\log \widehat{V_i}$ of large and medium QTLs as a function of the number of associated annotations ($h^2=0.5$, $k_\text {large}=1\%$; results for $h^2=0.5$, $k_\text {large}$ = 2.5% and 5% are shown in Additional file 1: Fig. S3). We first remark that the posterior variances of markers with smaller numbers of annotations tend to be underestimated. However, once a sufficient number of annotations are available (about 4 for large QTLs, and 7 for medium QTLs), estimated posterior variances approach the true simulated values. In this scenario, no QTLs overlap all 9 annotations, and several configurations of multi-annotations are possible, each with a different potential downstream impact. Thus, a marker included in two weakly enriched annotations is less likely to be assigned a strong or medium effect in the model, compared to one included in two highly enriched annotations.

#### Impact of directly modeling multi-annotated markers versus down-sampling annotations

**Fig. 4:**
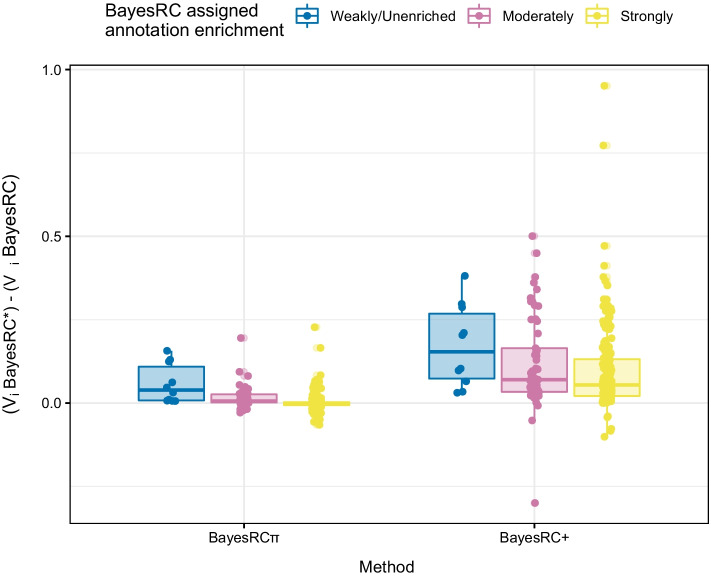
Impact of BayesRC random annotation assignment on large-effect QTL variance estimation. Boxplots represent the difference with BayesRC in estimated posterior variances for large-effect QTLs for BayesRC$\pi$ and BayesRC+. Results are shown according to the randomly assigned annotation categories (strongly, moderately, weakly/unenriched) used for BayesRC. All large QTLs across the 50 independent datasets are represented ($h^2=0.5$, $k_\text {large}=1\%$, scenario D with 9 annotations)

BayesRC$\pi$ shares greater similarity to BayesRC than BayesRC+, so its impact on prediction accuracy and marker variance estimation may potentially be more limited. However, assigning multi-annotated markers to a single annotation during data pre-processing, as we have done for BayesRC in this work, can have a negative effect on marker variance estimation. For example, inadvertently assigning a large-effect QTL to an uninformative annotation may lead to an underestimation of its effect. In Fig. 4 ($h^2=0.5$, $k_\text {large}=1\%$, scenario D), we show the difference in estimated posterior variance for BayesRC$\pi$ and BayesRC+ on the full set of large QTLs (250 across simulated datasets) with respect to BayesRC. We recall that in this scenario, all large QTLs were multi-annotated (systematically for the two strongly enriched annotations, often for moderately enriched annotations, and rarely for weak or unenriched annotations). Each large QTL was randomly assigned to a single annotation (unenriched, weakly, moderately, or strongly enriched) prior to fitting BayesRC, allowing an evaluation of the impact of these random assignments. As expected, estimated posterior variances are similar between BayesRC and BayesRC$\pi$ for large QTLs that were correctly randomly assigned to a strongly enriched annotation. However, those randomly assigned to a moderately enriched annotation saw an average gain of 0.016 (± 0.032), and a gain of 0.058 (± 0.058) for those erroneously assigned to a weakly enriched or unenriched annotation. By allowing multi-annotated markers to navigate among annotations, BayesRC$\pi$ thus avoids an underestimation of their effect related to an incorrect upstream assignment. A similar but stronger trend is observed for BayesRC+, though improved variance estimates are observed even for “correctly” assigned large QTLs. Average gains for BayesRC+ are 0.097 (± 0.13), 0.120 (± 0.14) and 0.171 (± 0.12) for large QTLs randomly assigned to highly, moderately, and weakly/unenriched annotations, respectively. Exploiting multiple annotations in an additive manner thus has a strong effect on variance estimation, in addition to compensating for potential misassignment of important QTLs to a less informative annotation.

#### Improved rankings of large-effect QTLs by posterior variances when incorporating annotations

One way to prioritize markers is to focus on those with the largest estimated posterior variances. Figure 4 suggests that BayesRC$\pi$ and BayesRC+ both yield larger estimated posterior variances to multi-annotated large QTLs than BayesRC; in turn, this tends to lead to a better average ranking for large QTLs (Table 2) for most simulation settings, especially for BayesRC+. One exception is the setting with $h^2=0.5$ and $k_\text {large}=5\%$, a favorable situation where BayesR readily prioritizes the simulated large QTLs without use of annotations. Otherwise in scenarios A, B and D, large QTL rankings are systematically improved in BayesRC$\pi$ compared to BayesRC, and in BayesRC+ compared to BayesRC$\pi$. Scenario C behaves somewhat differently, where large QTL rankings are generally worse and the best performing method depends on the settings. This is perhaps unsurprising, as scenario C is composed of the least informative annotation set. Overall, large QTL rankings for BayesRC and BayesRC$\pi$ are best in scenario A, followed by scenarios B and D; rankings for BayesRC+ appear to be more stable across scenarios for a given simulation setting. This robustness can likely be explained by the fact that BayesRC+ takes full advantage of the strongly enriched annotations present in scenarios A, B and D, while by design BayesRC$\pi$ may allow QTLs to be assigned to less enriched annotations in at least some iterations of the algorithm. This suggests that for the purposes of prioritizing QTLs, BayesRC$\pi$ may be more sensitive to the addition of noise in annotations.

**Table 2:** Average rankings of large QTLs by estimated posterior variance

Annotation scenario		None	A (2 annot.)			B (4 annot.)			C (4 annot.)			D (9 annot.)		
*h*^2^	*k*_*large*_ (%)	R	RC	RC$\pi$	RC+	RC	RC$\pi$	RC+	RC	RC$\pi$	RC+	RC	RC$\pi$	RC+
0.2	1	10286	501	479	**342**	652	617	**433**	4930	4446	**4212**	1268	1112	**322**
	2.5	2991	140	120	**91**	188	167	**110**	**1577**	1933	1755	340	303	**93**
	5	597	21	19	**14**	29	25	**18**	392	**361**	425	44	41	**16**
0.5	1	2711	162	140	**88**	194	152	**102**	1482	**1303**	1436	365	261	**102**
	2.5	127	12	11	**8**	21	12	**10**	**73**	74	104	23	20	**10**
	5	4	**3**	**3**	**3**	**3**	**3**	**3**	**4**	26	55	**3**	**3**	**3**

Mean rank (by decreasing estimated posterior variance) of large QTLs, averaged across 50 independent datasets for each setting (heritability, $k_\text {large}$) and each method (R = BayesR, RC = BayesRC, RC$\pi$ = BayesRC$\pi$ and RC+ = BayesRC+). With the exception of BayesR, which does not use annotations, results are presented by annotation scenario (A, B, C and D). Boldface is used to indicate the best ranking obtained for each setting. As each dataset contains 5 large QTLs, the highest average ranking that can be obtained is equal to 3 (average of 1 to 5)

### Genomic prediction results for pigs data

In the following, we illustrate the performance of our models on real data from a population of growing pigs, in conjunction with annotations related to multiple production traits from a public database.

#### Impact of pigQTLdb annotation strategies on prediction accuracy

**Fig. 5:**
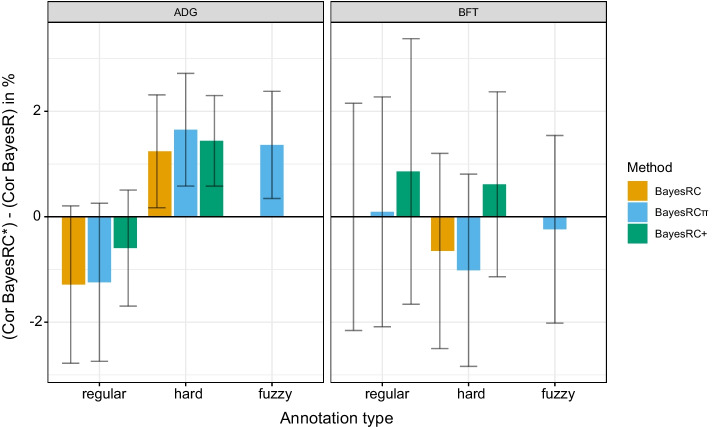
Differences in validation correlation with respect to BayesR for backcross pig data and various pigQTLdb annotations. Difference of correlation between the 3 methods including functional annotation (BayesRC, BayesRC$\pi$ and BayesRC+, gathered under the BayesRC* label) and BayesR, for average daily gain (ADG) and and back fat thickness (BFT) for three strategies of annotation construction: regular, hard, and fuzzy. Colored bars and error bars represent averages and standard deviations across 10-fold validation datasets

We first sought to determine whether the pigQTLdb annotations appear to contribute useful information for predicting BFT and ADG, and if so, whether the “hard” or “fuzzy” window-based annotations were beneficial (Fig. 5). As a baseline without annotations, BayesR yielded an average correlation of 0.21 (± 0.08) and 0.26 (± 0.16) for ADG and BFT, respectively. Two different behaviors are observed for model performance in the two traits when incorporating the pigQTLdb annotations. For ADG, prediction accuracy is deteriorated by the “regular” annotation (− 1.3, − 1.2, and − 0.6 correlation points for BayesRC, BayesRC$\pi$ and BayesRC+). However, by extending annotations to include the nearest neighboring markers (“hard”) led to respective gains of + 1.2, + 1.7, and + 1.4 points compared to BayesR. A similar gain in correlation (+ 1.4 points) was also achieved with BayesRC$\pi$ and “fuzzy” annotations allowing for an ambiguous neighborhood extension. For BayesRC$\pi$, we thus observe a difference of 2.9 correlation points between the “regular” and “hard” annotation strategies, highlighting the potential impact this upstream step plays. In the case of ADG, it is thus possible to identify a useful strategy for constructing and including annotation sets from pigQTLdb to improve trait prediction. On the contrary, for BFT these same pigQTLdb annotations appear to be less relevant for the task of prediction. For all annotation strategies, a largely equivalent or deteriorated prediction performance compared to BayesR is observed. Thus, for BayesRC, we go respectively from a zero average gain using “regular” annotations to a loss of − 0.6 points for “hard”. For BayesRC$\pi$, we go from a gain of + 0.1 points to a loss of − 1.0 points and − 0.2 points respectively with the “regular”, “hard” and “fuzzy” annotations. BayesRC+ presents the best results for this trait, with a gain of + 0.9 points and + 0.6 points for the “regular” and “hard” annotations; however, the results are highly variable, and appear to be insignificant for this trait.

#### PigQTLdb annotation category interpretation using BayesRC$\pi$

**Fig. 6:**
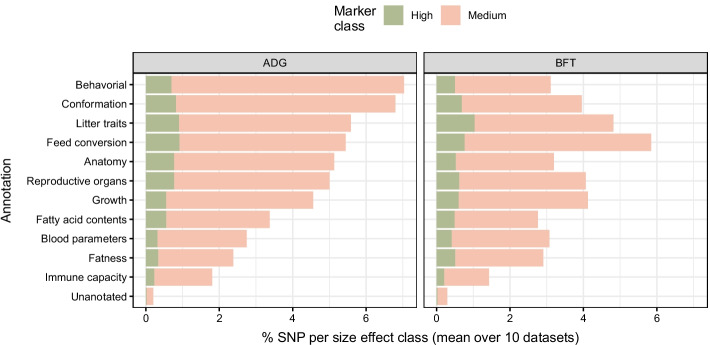
Interpretation of pigQTLdb annotations using BayesRC$\pi$ according to the trait for backcross pig data. For each annotation, we represent the proportion of markers assigned to the medium and large SNP effect size class, average on the 10-fold datasets. The two panels shows the results for the two studied trait, ADG and BFT. The order of annotation on y-axis is based on the decreasing value of the sum of large and medium proportion

In this study, we used sets of relatively common annotations from pigQTLdb without any upstream relevance selection; it may thus be of interest to evaluate the contribution of each to prediction using outputs from BayesRC$\pi$ (Fig. 6, “fuzzy” annotations). The average proportion of medium- or large-effect SNPs assigned to each annotation using the PAIP highlights a difference in estimated enrichment across annotation categories and between traits. For ADG, 7.0% of markers are classified as having medium or large effects in the Behavorial annotation compared to 1.8% for Immune capacity, i.e. almost 4 times less. The Unannotated category is made up of a large number of markers estimated to have null or small effects, and thus features few medium- to large-effect SNPs. Annotation category ranks were found to be identical when ordered by the median variance of assigned SNPs. Moreover, in the top 1% of markers (i.e., 469 SNPs, ranked by $\widehat{V_i}$), more than half were assigned to the Anatomy (21%), Behavorial (17%) and Conformation (15%) annotations. Conversely, Immune capacity represents only 0.4% of these top SNPs, less than the Unannotated category (1.4%). Taken together, we can for example question the relevance of the Immune capacity annotation for predicting this trait.

With respect to BFT, given the poor prediction quality observed for all annotation strategies (Fig. 5), we must interpret the relevance of annotations with caution. The most enriched annotation for BFT is Feed conversion, while Behavorial drops from the first position in ADG to the 7th here. Immune capacity again shows very low enrichment (1.4%). In general, the annotations appear to be more weakly enriched in BFT (3.5% of markers in medium to high classes) than in ADG (4.5%). On the contrary, the enrichment of the Unannotated category increases slightly in BFT (0.3%) compared to ADG (0.2%), suggesting the possibility of important markers for this trait being unannotated in PigQTLdb.

#### Fuzzy and hard expanded windows for annotation construction

The “fuzzy” strategy we have explored here is essentially best adapted to BayesRC$\pi$, as it allows for ambiguity in annotation assignment by design. In the case of ADG, this annotation strategy provided good predictions. To give additional insight into the behavior of BayesRC$\pi$ for “fuzzy” annotations, we provide an illustration for ADG in Additional file 1: Fig. S5 of the posterior variance of markers, averaged over the ten cross-validation datasets, according to their relative position (zoomed in between 8000 and 12000 for clarity). We note that a large discontinuous block of markers were directly annotated within pigQTLdb. We focus our attention on markers with ambiguous annotations (i.e., those neighboring the pigQTLdb markers, and thus included with annotations in the “hard” and “fuzzy” strategies). These “ambiguously annotated” SNPs systematically have larger estimated values for $\widehat{V_i}$ in both the “fuzzy” and “hard” strategies. For example, the marker at relative position 11554 (which is a direct neighbor of a pigQTLdb-annotated marker) is estimated to have a posterior variance of 0.85 with the “regular” annotation, 16.81 with “fuzzy” and 24.78 with “hard”. To avoid overestimating the effect of unimportant neighboring markers, the “fuzzy” strategy allows for their potential assignment to the Unannotated category, thus giving them less chance to be estimated as non-null in the model. This dampening effect can be seen in Additional file 1: Fig. S5, where some of the ambiguously annotated markers have estimated posterior variances intermediate to those the unannotated and annotated markers.

#### Comparison of top ranked SNPs by estimated posterior variance

**Fig. 7:**
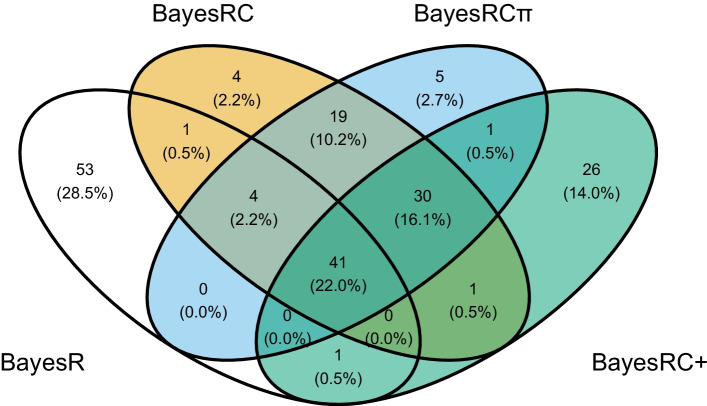
Top-ranked SNPs by estimated posterior variance in backcross pig data. Venn diagram of the lists of 100 most highly ranked SNPs by estimated posterior variance for each Bayesian method, averaged across ten cross-validation datasets

Incorporating biological information in genomic prediction models has the potential to improve their interpretability by better prioritizing informative markers. In a similar spirit to genome-wide association studies (GWAS), where markers are prioritized by *p*-value from a univariate test of association, we can use the estimated posterior variances from the Bayesian models to rank markers. To evaluate the similarities and differences in top-ranked SNPs for each method, we selected for each the top 100 markers according to the average (across 10 cross-validation datasets) estimated posterior variances. Results are shown in Fig. 7 for ADG and “hard” pigQTLdb annotations (a setting with good prediction results) for BayesRC, BayesRC$\pi$ and BayesRC+; BayesR, without annotations, is also included for reference. 41 markers were ranked in the top 100 by all methods, with most (40 markers) included in at least one annotation. More than half (53 %) of the markers prioritized by BayesR were not highly ranked by the other methods, including 31 unannotated markers. On the other hand, unsurprisingly all 30 markers prioritized by BayesRC, BayesRC$\pi$ and BayesRC+ were annotated. Finally, our newly proposed models BayesRC$\pi$ and BayesRC+ highly ranked 32 markers not highly ranked by the others. Of these, all of which were annotated, 18 featured multiple annotations: 8 markers with 2 annotations, 3 with 3 annotations, 2 with 5 annotations and 1 with 7 annotations.

## Discussion

In this work, we presented two new genomic prediction methods (BayesRC$\pi$ and BayesRC+) that allow for the use of multiple, overlapping annotations, which until now has been a limiting factor in the BayesRC reference method. This led us to compare the three methods for integrating annotations in simulated and real data with different genomic architectures and sets of annotations. To evaluate the interest of adding annotation information to genomic prediction models, we used BayesR as a reference. We constructed a variety of simulations to study the impact of favorable and unfavorable annotation sets and identify appropriate use cases for each of the proposed models. Since BayesRC$\pi$ operates by stochastically classifying multi-annotated markers to a single annotation, we often observed little difference with BayesRC. We remark that building relevant and irrelevant annotations in our simulations was not a straightforward task, as the incorporation of a single large QTL (or a single marker in LD with a large QTL) de facto changes the enrichment of a given annotation. Thus, large QTLs were mostly multi-annotated for highly or moderately enriched annotations, a favorable situation for BayesRC despite the random assignment we used. It is also important to note that if annotations contain few overlaps, differences with BayesRC would be further reduced for both BayesRC$\pi$ and BayesRC+. However, the annotation scenarios we considered in our simulation study nevertheless allowed us to highlight the behavior of BayesRC$\pi$ and BayesRC+ in a variety of situations.

In addition to the simulated data, we illustrated our approaches on data from a backcross population of growing pigs in conjunction with annotations from pigQLTdb. All available annotations were used for two traits (ADG and BFT) to study differences in model behavior. As for the simulations, we observed situations where the use of annotations was not advantageous, as was the case for BFT. Another choice that was decisive in the prediction accuracy of the models was the sibling-structured cross-validation procedure we used. Animals were grouped into ten sets by their fathers, meaning that animals within the validation set tended to be genetically similar to one another, and potentially distant from the training data. This necessarily complicates the prediction task compared to a fully randomized cross-validation procedure. It has been previously suggested that the use of annotations in situations where the validation population is genetically distant from the learning population could lead to improvement; this holds on average for the ADG trait when appropriate annotations are used.

The optimal use of our proposed models appears to depend on several factors, including the genomic architecture, the relevance and construction strategy of annotations, the number and interpretation of overlapping annotations, and the desired goal. There were in fact cases where BayesR (without annotations) yielded better results than models including annotations. These likely correspond to situations where annotations are not relevant to the trait of interest. In such cases, BayesRC$\pi$ provides tools to interpret the relevance of annotations a posteriori using the PAIP statistic. On the other hand, although its assumption of additivity may be overly strong in some cases, BayesRC+ tends to compensate for the underestimation of QTL posterior variances and encourages a prioritization of multi-annotated markers. As such, for BayesRC+ it is important that overlapping annotations represent not uncertainty but rather complementary information leading to greater confidence in the impact of SNPs on the phenotype.

Via the “regular”, “hard” and “fuzzy” window annotation strategies, we saw that there are cases where it may be interesting to expand annotations to include neighboring markers. This is likely linked to uncertainty of the precise location of causal mutations and as well as LD structures with nearby markers, especially when SNP density is low to medium (under 100K) and/or LD extent is large (familial structures). The fuzzy annotation strategy represents a potentially interesting approach to capitalize on the modeling specificity of BayesRC$\pi$, as it provides indicators of the relevance of multiple annotations. It could also be potentially interesting to use BayesRC$\pi$ outputs to refine and adapt the annotation set, for example by merging, splitting or deleting some annotations. A more exhaustive exploration of different strategies for constructing annotations would be a useful avenue for future research.

The methods we have proposed here take into account annotations coded as binary classifications, and thus do not reflect the potentially continuous nature of underlying annotations (e.g., GWAS *p*-values). It would therefore be interesting to extend these approaches to handle continuous annotations, in particular to allow the flexibility to give greater weight to certain markers in annotations, or to certain annotations for a given marker. This future development has the potentially to more fully exploit the heterogeneous and complex functional information that is increasingly available.

## Conclusions

The full use of complex and potentially overlapping annotations can improve genomic prediction and the estimation of posterior variance for markers. These annotations impact the results of the different prediction methods used, and their use must make sense with respect to the studied trait. We proposed two new methods based on different assumptions on the interpretation of multi-annotated markers: allowing such SNPs to be assigned to the most representative annotation (BayesRC$\pi$) or cumulatively assigning a greater weight for such SNPs (BayesRC+). These models each perform well in different settings, and lead to different downstream analyses. We observed average gains of up to 2 correlation points compared to a model ignoring annotations in simulated data, and up to 1.7 points on real data. Models integrating annotations, and in particular BayesRC$\pi$ and BayesRC+, are particularly good at prioritizing medium and large QTLs according to their estimated posterior variances. BayesRC$\pi$ and BayesRC+, in addition to BayesC$\pi$, BayesR and BayesRC, have been implemented in an open-source software package called *BayesRCO*. Many strategies for constructing annotations are possible, and we compared several approaches based on extended hard or fuzzy windows around known pigQTLdb hits. In future work, a promising approach would be to further extend our models to fully account for continuous, rather than categorical, annotations.

## Supplementary Information

**Additional file 1: Figures S1–S5** and pseudocode for the BayesR, BayesRC, BayesRC$\pi$, and BayesRC+ algorithms.

## Data Availability

We implemented the two novel methods BayesRC$\pi$ and BayesRC+ in a Fortran software package named *BayesRCO* (BayesRC for Overlapping annotations), available at https://github.com/fmollandin/BayesRCO, https://doi.org/10.5281/zenodo.6809653. In addition, the package also implements BayesC$\pi$, BayesR, and BayesRC. The associated GitHub repository includes a full user’s guide, detailing the different types of parameterization possible. Code used to simulate phenotypes based on genotype data is available in the “Simulation” folder of the following Github repository: https://github.com/fmollandin/BayesR_Simulations. An R script to extract and format pigQTLdb annotations is available in the following repository: https://github.com/andreamrau/tidyqtldb.
