# Supplementary material for: Accounting for overlapping annotations in genomic prediction models of complex traits
Source: BMC Bioinformatics. 2022 Sep 6;23:365. doi: 10.1186/s12859-022-04914-5 (PMC9446854; doi:10.1186/s12859-022-04914-5)
Supplement: Supplementary file 1 — Additional file 1: Figures S1–S5 and pseudocode for the BayesR, BayesRC, BayesRC$\pi$π, and BayesRC+ algorithms. [file 12859_2022_4914_MOESM1_ESM.pdf]

# Supplementary Materials: Accounting for overlapping annotations in genomic prediction models of complex traits

Fanny Mollandin[1], Hélène Gilbert[2], Pascal Croiseau[1], and Andrea Rau[1,3]

[1] Université Paris-Saclay, INRAE, AgroParisTech, GABI
[2] GenPhySE, Université de Toulouse, INRAE, ENVT
[3] BioEcoAgro Joint Research Unit, INRAE, Université de Liège, Université de Lille, Université de Picardie Jules Verne

**S1 Supplementary Figures** **S2**

**S2 Algorithm pseudocode and details** **S7**

# S1 Supplementary Figures

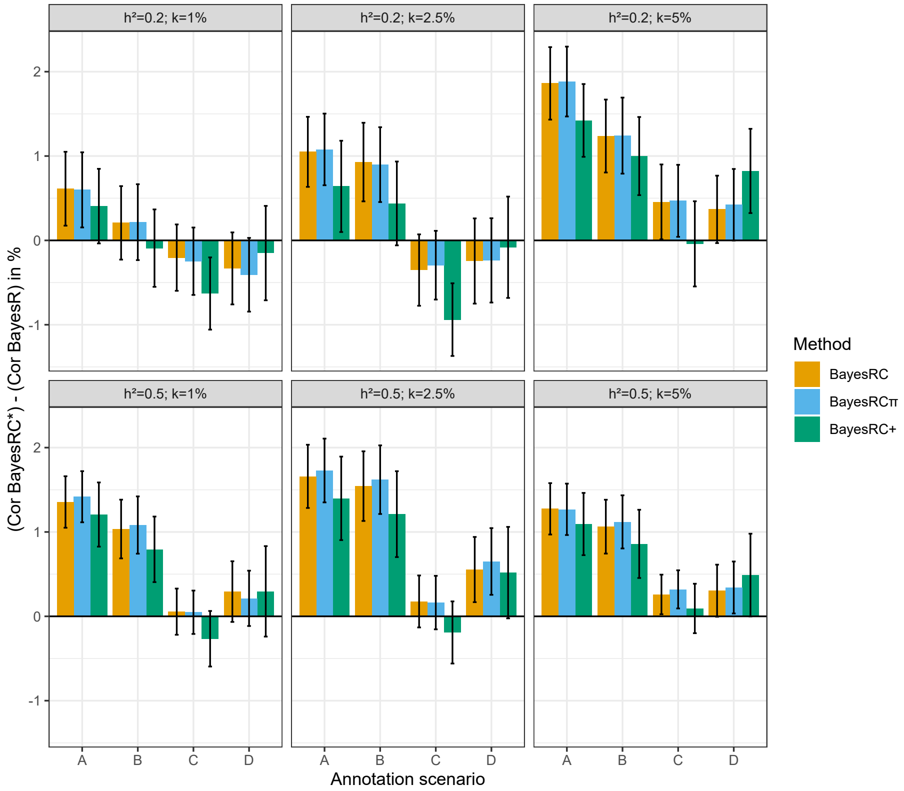


Supplementary Figure S1: Differences in validation correlation with respect to BayesR for four annotation scenarios. For $h^2 = 0.2$ and $h^2 = 0.5$ and $k_{\text{large}} = 1\%$, $k_{\text{large}} = 2.5\%$ and $k_{\text{large}} = 5\%$, the difference in validation correlation between the three models including annotations (BayesRC, BayesRC$\pi$ and BayesRC+, gathered under the BayesRC* label) and BayesR, which does not include annotations. Colored bars and error bars represent averages and the 95% confidence interval across 50 simulated datasets.

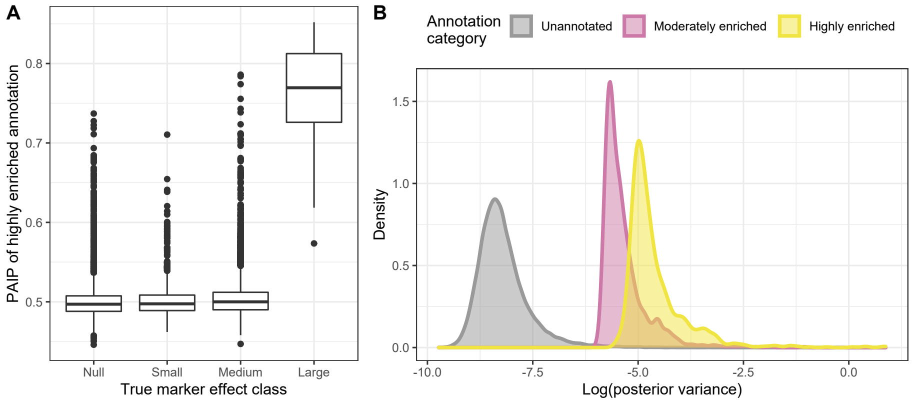


Supplementary Figure S2: Using the PAIP to interpret annotation importance for BayesRC$\pi$. Results are shown for $h^2 = 0.5$, $k_{\text{large}} = 5\%$, and scenario A. (A) Posterior mean frequency of marker assignment to the strongly enriched annotation (i.e., strongly enriched PAIP) by simulated effect size category (null, small, medium, high). Results are averaged across 50 independent datasets. (B) Distribution of the log posterior variance of markers by PAIP-assigned annotation for one illustrative dataset.

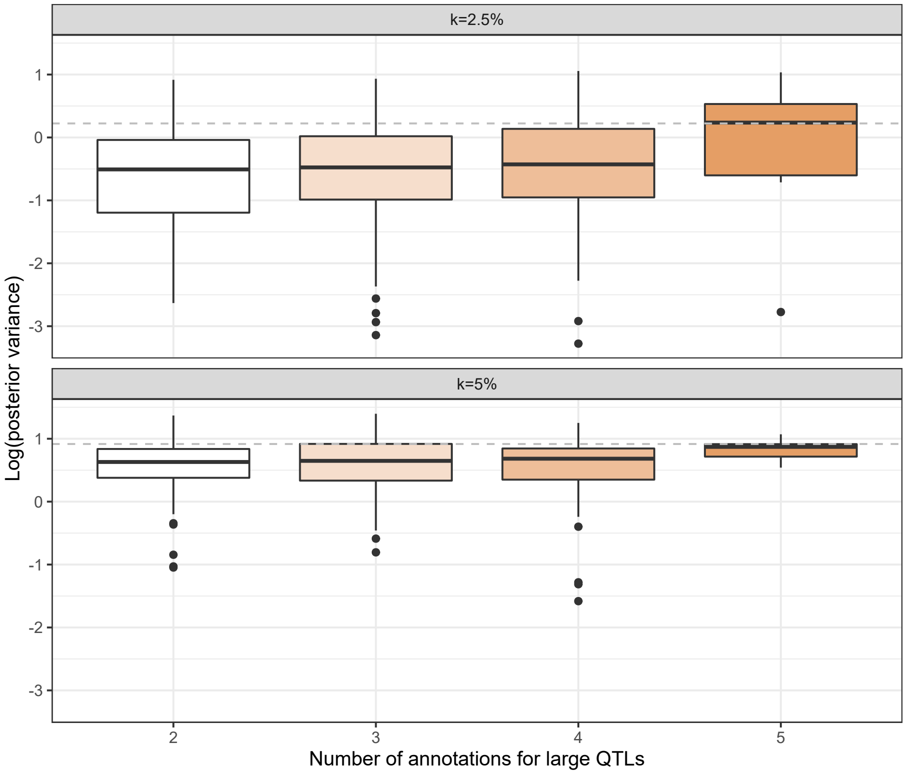


Supplementary Figure S3: Impact of number of annotations on markers for BayesRC+ model. Log posterior variance of large effect QTLs by the number of associated annotations. All QTLs across the 50 independent datasets are represented. Results are shown for $h^2 = 0.5$ and scenario D (including 9 annotations), and for $k_{\text{large}} = 2.5\%$ (top panel) and $k_{\text{large}} = 5\%$ (bottom panel). The black dotted lines represent the true simulated value of $\log V_i$ for each $k_{\text{large}}$.

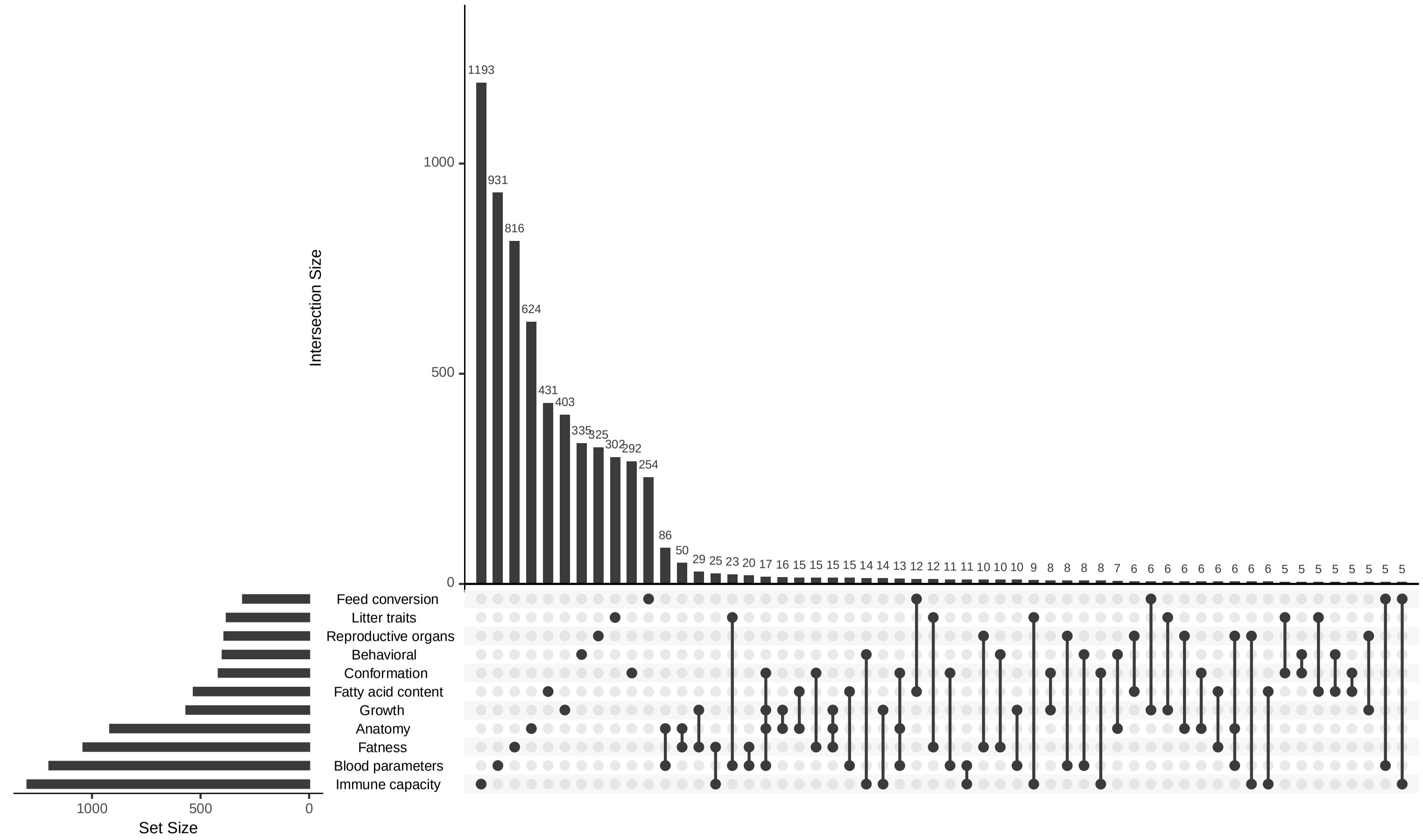


Supplementary Figure S4: pigQTLdb annotation structure. Upset plot of the 11 pigQTLdb annotations, representing their respective numbers and the overlaps between annotations. For the purpose of visibility, only overlaps between annotations concerning more than 5 markers have been represented.

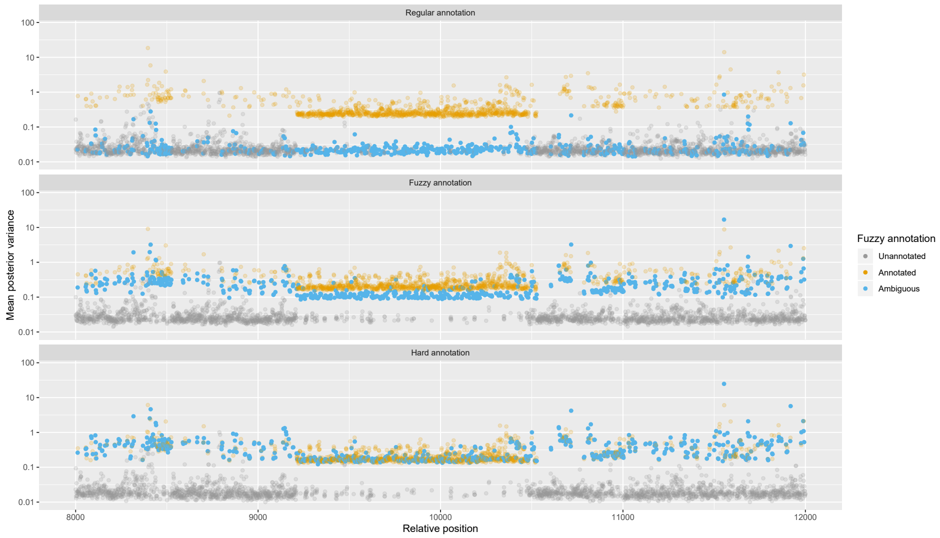


Supplementary Figure S5: Impact of annotation construction on variance estimation in BayesRC$\pi$ for ADG trait. The three panels correspond to “regular” (top), “fuzzy” (middle), and “hard” (bottom) annotations for BayesRC$\pi$. For each, the posterior variance of markers, averaged on the 10-fold datasets, is represented and colored according to its fuzzy window annotation: unannotated, annotated or ambiguously annotated (i.e both annotated and unannotated).

## S2 Algorithm pseudocode and details

**Algorithm 1** BayesR pseudo-code

```
Initialization
for each iteration do
    update μ|.
    for i in 1:p do
        ỹ = ỹ + x_{.,i} β_i
        for k in 1:4 do
            compute LogL(i, k|π)
        end for
        assign SNP i to k̂ ∈ {1, 2, 3, 4} |LogL(i, .|π)
        update β_i|k̂
        ỹ = ỹ − x_{.,i} β_i
    end for
    update σ_g^2, σ_e^2, π
end for
```

**Algorithm 2** BayesRC pseudo-code; $\#C_i = 1\ \forall i$

```
Initialization
for each iteration do
    update μ|.
    for i in 1:p do
        ỹ = ỹ + x_{.,i} β_i  c = C_i
        for k in 1:4 do
            compute LogL(i, k|c, π_c)
        end for
        assign SNP i to k̂ ∈ {1, 2, 3, 4} |LogL(i, .|c, π_c)
        update β_i|k̂
        ỹ = ỹ − x_{.,i} β_i
    end for
    update σ_g^2, σ_e^2, π_{c_1}, π_{c_2}, ...π_{c_m}
end for
```

**Algorithm 3** BayesRC$\pi$ pseudo-code; $\#C_i \geq 1\ \forall i$

Initialization
**for** each iteration **do**
    update $\mu|.$
    **for** i in 1:p **do**
        **for** $c \in C_i$ **do**
            compute $LogL(i, c|p_i)$
        **end for**
        assign SNP $i$ to $\hat{c} \in C_i\ |LogL(i, .|p_i)$
        $\tilde{y} = \tilde{y} + x_{.,i}\beta_i$
        **for** k in 1:4 **do**
            compute $LogL(i, k|\hat{c}, \pi_{\hat{c}})$
        **end for**
        assign SNP $i$ to $\hat{k} \in \{1, 2, 3, 4\}\ |LogL(i, .|\hat{c}, \pi_{\hat{c}})$
        update $\beta_i|\hat{k}$
        $\tilde{y} = \tilde{y} - x_{.,i}\beta_i$
    **end for**
    update $\sigma_g^2, \sigma_e^2, \pi_{c_1}, \pi_{c_2}, ...\pi_{c_m}, p_1, p_2, ..., p_p$
**end for**

**Algorithm 4** BayesRC+ pseudo-code; $\#C_i \geq 1\ \forall i$

Initialization
**for** each iteration **do**
    update $\mu|.$
    **for** i in 1:p **do**
        $\tilde{y} = \tilde{y} + x_{.,i}\beta_i$
        **for** $c \in c_i$ **do**
            **for** k in 1:4 **do**
                compute $LogL(i, k|c, \pi_c)$
            **end for**
            assign SNP $i$ to $\hat{k} \in \{1, 2, 3, 4\}\ |LogL(i, .|c, \pi_c)$
            update $\beta_{i,c}|\hat{k}$
            $\tilde{y} = \tilde{y} - x_{.,i}\beta_{i,c}$
        **end for**
        $\beta_i = \sum_{c \in c_i} \beta_{i,c}$
    **end for**
    update $\sigma_g^2, \sigma_e^2, \pi_{c_1}, \pi_{c_2}, ...\pi_{c_m}$
**end for**
